# Evaluation of various feedstuffs of ruminants in terms of chemical composition and metabolisable energy content

**DOI:** 10.14202/vetworld.2015.605-609

**Published:** 2015-05-14

**Authors:** Dinesh Kumar, Chander Datt, L. K. Das, S. S. Kundu

**Affiliations:** 1 Department of Animal Nutrition, Centre of Advanced Faculty Training in Animal Nutrition, Indian Veterinary Research Institute, Izatnagar, Uttar Pradesh, India; 2 Division of Dairy Cattle Nutrition, National Dairy Research Institute, Karnal, Haryana, India; 3 Veterinary Dispensary, Kalampur, Kalahandi, Odisha, India

**Keywords:** chemical compositions, feedstuffs, *in vitro* method, metabolisable energy, ruminants

## Abstract

**Aim:**

The aim was to determine the chemical composition and metabolisable energy (ME) content of feedstuffs used in ruminant animals using *in vitro* method.

**Materials and Methods:**

A total of 18 feedstuffs used for ruminant feeding including cultivated non-leguminous fodders like maize, sorghum, pearl millet, and oat; leguminous fodders like cowpea and berseem; agro-industrial by-products such as wheat bran, deoiled rice bran, rice polish, wheat straw, and concentrates such as mustard oil cake, groundnut cake, soybean meal, cotton seed cake, grains like maize, oat, wheat, and barley were taken for this study. Chemical compositions and cell wall constituents of test feeds were determined in triplicate. The crude protein (CP) content was calculated as nitrogen (N) × 6.25. True dry matter digestibility (TDMD), true organic matter digestibility (TOMD), ME, and partitioning factor (PF) values were determined by *in vitro* gas production technique (IVGPT).

**Results:**

The CP content of non-leguminous fodders varied from 7.29% (sorghum) to 9.51% (maize), but leguminous fodders had less variation in CP. Oilseed cakes/meals had high CP and ether extract (EE) content than other feedstuffs except rice polish, which had 12.80% EE. Wheat straw contained highest fiber fractions than the other ingredients. ME content was highest in grains (wheat-12.02 MJ/kg) and lowest in wheat straw (4.65 MJ/kg) and other roughages. TDMD of grains and oilseed cakes/meals were higher than the fodders and agro-industrial by-products. The same trend was observed for TOMD.

**Conclusions:**

It was concluded that the energy feeds showed a great variation in chemical composition and ME content. The results of this study demonstrated that the kinetics of gas production of energy feed sources differed among themselves. Evaluation of various feedstuffs is helpful in balanced ration formulation for field animals and under farm conditions for better utilization of these commonly available feed resources.

## Introduction

India has a very large population of livestock both of productive and unproductive animals. Ruminants are mostly fed on low-quality roughages, which are poor in protein, energy, minerals, and vitamin contents. Addition of cereal and leguminous fodder in ruminant diets can improve the utilization of low-quality roughages mainly through the supply of N to rumen microbes. The ruminants can make efficient use of mill by-products, crop residues, and other non-conventional feed sources being equipped with rumen microbial ecosystem. The majority of livestock is fed a very small quantum of concentrates and productive animals are fed either balanced concentrate or raw ingredients, which are traditionally mixed on farms and which do not meet the complete nutrient requirements of livestock and hence can be called unbalanced [1]. Hence, knowledge regarding the nutrient composition of different feedstuffs helps in preparation of balanced rations for ruminants. Database on nutritive values, access, and use of such information will have a significant impact on improved animal performance and productivity [2]. The nutritive value of ruminant feed is determined by the concentration of its chemical compositions, as well as rate and extent of digestion in the rumen. The ultimate goal of feed analysis is to predict the productive response of animals when they are fed rations of a given composition. This is the real reason for the necessity of information on feedstuff composition.

There are different methods available to ascertain the nutritive worth of feedstuffs. Though conducting *in vivo* trials seems to be the best method however, they are time consuming, laborious, expensive, require large quantities of feed, and are not suitable when evaluation of large number of feeds are involved [3]. Correct assessment of the energetic density of a diet is fundamental to accurate prediction of productive performance in dairy cows [4]. *In vitro* gas production (IVGP) is an alternative technique used to determine the nutritive value of feedstuffs, since rate and extent of degradation, and rumen fermentation can be easily determined by measurement of cumulative gas production [5]. Datt *et al*. [6] also used the *in vitro* gas production technique (IVGPT) to evaluate nutritive values of leguminous and non-leguminous crops. *In vitro* trials, on the other hand, are less time-consuming, cheaper and more efficient when large number of samples are to be handled.

Therefore, the objective of this study was to generate information on nutritional quality of locally available feeds or introduced fodders, so that a data bank could be established which would be helpful in ration formulation for enhancing ruminant productivity. Thus, the feedstuffs available in and around Karnal, Haryana were evaluated for various nutritional aspects in terms of chemical compositions, true dry matter digestibility (TDMD)/true organic matter digestibility (TOMD), and metabolisable energy (ME) values.

## Materials and Methods

### Ethical approval

The experimental design and plan of the present study strictly followed the norms of Institutional Animal Ethics Committee (IAEC) of National Dairy Research Institute (NDRI), Karnal, Haryana. The present study primarily involved laboratory analysis of ruminant feeds, for which requisite permission was granted to collect rumen liquor from fistulaed animal by academic council and IAEC of NDRI, Karnal, Haryana.

### Sample collection

Various types of feedstuffs used for ruminant feeding including cultivated non-leguminous fodders like maize (*Zea mays*), sorghum (*Sorghum bicolor*), pearl millet (*Pennisetum typhoides*) and oat (*Avena sativa*); leguminous fodders like cowpea (*Vigna unguiculata*) and berseem (*Trifolium alexandrinum*), agricultural by-products such as wheat bran, deoiled rice bran (DORB), rice polish, wheat straw, concentrates like mustard oil cake (MOC) (*Brassica juncea*), groundnut cake (GNC) (*Arachis hypogaea*), soybean meal (SBM) (*Glycine max*), cotton seed cake (CSC) (*Gossypium* spp.), and grains like maize, oat, wheat (*Triticum aestivum*), and barley (*Hordeum vulgare*) were collected in and around Karnal, Haryana. The samples were oven dried at 70°C for overnight, ground to pass through 1 mm sieve and stored in sampling bottles.

### Chemical analyses

The feed samples were subjected to proximate analysis (crude protein [CP], ether extract (EE) and ash) as per the standard procedures of AOAC [7] in triplicate. The CP content was calculated indirectly by multiplying a factor of 6.25 to the estimated Nitrogen (N) content (N × 6.25). The cell wall constituents (neutral detergent fiber (NDF), acid detergent fiber (ADF), lignin, cellulose, and hemicellulose) of feeds were estimated by the methods of Goering and Van Soest [8].

### *In vitro* gas production method

*In vitro* methods are widely used to evaluate the nutritive value of different classes of feeds [9,10]. *In vitro* trials were conducted in triplicate to estimate various parameters such as total gas production, *in vitro* dry matter/organic matter (DM/OM) digestibility, metabolizable energy (ME), and partitioning factor (PF). These trials were conducted along with respective blank in triplicate. The incubations were carried out in 100 ml calibrated glass syringes (Fortuna Optima, Germany) as described by Menke and Steingass [11]. The syringes were incubated in a water bath at 39±0.5°C [12]. The substrate was weighed on a plastic boat with removable stem and was placed into the bottom of the plastic syringe without sticking to the sides of the syringe. The piston was lubricated with petroleum jelly and pushed inside the glass syringe. The syringes were kept in an incubator at 39°C until incubation.

The medium mixture solution was prepared by mixing 500 ml distilled water, 0.125 ml micro minerals solution, 250 ml rumen buffer solution and 250 ml macro minerals solution, 1.25 ml resazurin solution, and 50 ml reducing solution (prepared fresh and added just prior to incubation). The medium mixture solution was pre-warmed to 39°C and bubbled with CO_2_ just before collection of rumen liquor.

Rumen liquor was collected from a fistulated Sahiwal adult male cattle (age: 5-6 year, body weight: 470 kg) maintained on a standard diet (60 parts roughage: 40 parts concentrate) before morning feeding and watering into a pre-warmed thermos flask and brought to the laboratory. The donor animal was fed according to its requirements [13]. The rumen liquor was filtered through four layers of muslin cloth and pooled together which was used as inoculum source [14]. Once the medium mixture solution became colorless or reduced, the required amount of filtered rumen liquor was added. According to Mertens *et al*. [15], reduction of buffer prior to addition of the rumen inoculums was extremely important to minimize the shock to strictly anaerobic micro-organisms, this would in turn reduced the resulting lag phase experienced in terms of substrate degradation. The proportion of medium mixture solution to rumen liquor was 2:1. Just after mixing the medium and rumen liquor, 30 ml of the incubation medium was injected to the syringes using auto dispenser. The syringes were shaken gently and residual air or air bubbles, if any, was removed and outlet was closed. The gas measurements were carried out at every 4 h interval after incubation. The cumulative gas production values (up to 24 h) and values of other chemical constituents (CP, EE, and ash) were used to arrive at the feed ME values using the equation given by Menke *et al*. [16]. *In vitro* DM and OM digestibility were estimated using methods of Van Soest *et al*. [17].

### Statistical analysis

The results obtained during this study were analyzed by using software package SPSS version 16.0 [18].

### For concentrate feeds (grains, oil seed cakes/meals, and by-products)

ME (MJ/kg DM) = 1.06 + 0.1570 × Gas produced (ml/200 mg DM) + 0.0084 × CP (g/kg DM) + 0.022 × EE (g/kg DM) – 0.0081 × Ash (g/kg DM).

### For roughage feeds (green fodders)

ME (MJ/kg DM) = 2.20 + 0.1357 × Gas Produced (ml/200 mg DM) + 0.0057 × CP (g/kg DM) + 0.0002859 × EE² (g/kg DM).

## Results and Discussion

The proximate composition of various feedstuffs has been presented in Table-1. The cultivated fodders included those, which are grown locally like leguminous and non-leguminous fodders. DM and CP contents of different fodders showed wide variations. These variations could be a result of agronomic factors such as application of various levels of N fertilizers, time of harvest, ensiling, field drying, and storage. The CP content of non-leguminous fodders varied from 7.29% (sorghum) to 9.51% (maize), but leguminous fodders have less variation in CP (16.58% in cowpea and 17.05% in berseem). Oilseed cakes/meals had high CP and EE than other feedstuffs except rice polish, which had 12.04% EE. All grains had similar CP values. All agro-industrial by-products had higher CP content except wheat straw, which recorded minimum CP content (3.39%) among all feeds.

**Table-1:** Proximate composition of feedstuffs (% DM).

Feed	DM	OM	CP	EE	Total ash
Grains					
Maize	92.71±0.33	96.99±0.02	9.51±0.32	4.43±0.24	3.01±0.02
Oat	89.99±0.38	96.82±0.04	9.82±0.40	4.51±0.12	3.18±0.04
Wheat	92.00±0.15	97.68±0.10	11.71±0.14	2.02±0.03	2.32±0.10
Barley	92.66±0.33	96.07±0.25	10.18±0.32	1.69±0.06	3.93±0.25
Oilseed cakes/meals					
GNC	93.18±0.64	94.22±0.18	41.54±0.22	6.88±0.09	5.78±0.18
MOC	92.77±0.09	94.89±0.26	37.29±0.19	7.03±0.06	5.11±0.26
CSC	91.30±0.28	95.56±0.19	27.11±0.28	7.51±0.04	4.44±0.19
SBM	91.41±0.61	93.37±0.87	45.59±0.25	2.01±0.09	6.63±0.87
Agro-industrial by-products					
Wheat bran	91.33±0.45	94.24±0.08	14.46±0.48	3.14±0.09	5.76±0.08
DORB	92.33±0.70	90.63±0.07	13.25±0.21	1.81±0.17	9.37±0.07
Rice polish	88.50±0.15	86.45±0.22	12.80±0.07	12.04±0.29	13.55±0.22
Wheat straw	90.13±0.21	90.21±0.07	3.39±0.16	1.04±0.02	9.79±0.07
Leguminous green fodders					
Cowpea	16.58±0.34	91.04±0.19	17.74±0.21	2.95±0.10	8.96±0.19
Berseem	17.05±0.42	89.91±0.22	17.90±0.31	2.83±0.10	10.09±0.22
Non-leguminous green fodders					
Maize	15.96±0.99	88.64±0.13	9.51±0.19	1.36±0.07	11.36±0.13
Oat	11.39±0.25	88.03±0.41	8.80±0.16	2.74±0.12	11.97±0.41
Sorghum	18.81±0.78	88.09±0.28	7.29±0.06	1.81±0.08	11.91±0.28
Pearl millet	20.57±0.49	89.95±0.05	8.07±0.18	2.91±0.08	10.05±0.05

DM=Dry matter, OM=Organic matter, CP=Crude protein, EE=Ether extract, GNC=Groundnut cake, MOC=Mustard oil cake, CSC=Cotton seed cake, SBM=Soybean meal, DORB=Deoiled rice bran

Cell wall composition of feedstuffs is shown in the Table-2. Wide variations in the cell wall composition of all feeds were observed. Wheat straw contained highest NDF and ADF among all feeds. Higher fiber and lower protein content of wheat straw demonstrated its poor nutritional quality. Leguminous fodders showed comparatively lower NDF contents than that of non-leguminous fodders. Together with higher protein content, leguminous fodders were found to be of better feeding value for ruminants. Grains contained lower NDF values except barley, which might be due its tough fibrous seed coat. Similarly, CSC among oilseed cakes/meals showed higher NDF content, most probably due to the process of un-decortication of cotton seeds during oil extraction. Lignin content was found to be higher in feeds like leguminous fodders, agro-industrial by-products (rice polish and wheat straw), and CSC. Higher lignin content of feeds usually interferes with fiber availability to ruminants.

**Table-2:** Cell wall constituents of feedstuffs (% DM).

Feed	NDF	ADF	Hemicellulose	Cellulose	Lignin
Grains					
Maize	26.15±0.11	6.64±0.23	19.51±0.21	4.91±0.26	1.50±0.21
Oat	27.05±0.33	9.05±0.21	18.00±0.12	6.42±0.33	2.58±0.08
Wheat	19.71±0.08	5.75±0.20	13.96±0.22	3.61±0.53	1.65±0.03
Barley	42.05±0.87	10.77±0.23	31.28±0.69	6.08±0.10	3.05±0.26
Oilseed cakes/meals					
GNC	24.17±0.61	18.47±0.63	5.70±0.51	13.59±0.12	3.59±0.03
MOC	23.33±0.36	17.73±0.74	5.60±0.40	13.77±0.59	3.23±0.05
CSC	47.31±0.88	35.56±0.72	11.75±0.70	24.40±0.66	7.26±0.07
SBM	18.23±0.29	10.47±0.26	7.76±0.19	8.40±0.18	1.85±0.13
Agro-industrial by-products					
Wheat bran	41.16±0.41	12.57±0.21	28.59±0.24	9.15±0.15	2.92±0.17
DORB	47.16±0.71	16.98±0.35	30.18±0.13	10.15±0.16	5.42±0.22
Rice polish	38.22±0.34	21.01±0.53	17.21±0.20	12.62±0.42	7.01±0.14
Wheat straw	76.22±0.98	50.99±0.44	25.23±0.71	36.07±0.55	9.11±0.06
Leguminous green fodders					
Cowpea	47.10±1.29	33.06±0.24	14.04±1.47	19.81±0.32	8.80±0.89
Berseem	43.28±0.66	24.08±0.52	19.20±0.16	15.04±0.16	7.09±0.13
Non-leguminous green fodders					
Maize	66.69±0.42	39.67±0.26	27.03±0.65	32.53±1.51	5.49±0.27
Oat	53.25±0.33	27.41±0.19	25.84±0.44	20.22±0.37	3.73±0.09
Sorghum	70.01±0.61	42.25±0.44	27.76±0.99	35.88±0.72	4.33±0.23
Pearl millet	63.63±1.40	37.86±0.57	25.77±1.78	30.13±0.55	5.18±0.30

NDF=Neutral detergent fiber, ADF=Acid detergent fiber, DM=Dry matter, GNC=Groundnut cake, MOC=Mustard oil cake, CSC=Cotton seed cake, SBM=Soybean meal

The IVGP, ME content, DM and OM digestibility and PF of various feedstuffs has been presented in Table-3. In general, predicted ME values were very low in the feedstuffs having high fiber and low protein contents. Lower ME value (4.65 MJ/kg) was found in wheat straw. These roughages are deficient in fermentable carbohydrates, reflected by relatively low OM digestibility [19]. There did not seem appreciable differences among cultivated leguminous and non-leguminous fodders for TDMD/TOMD and ME content. The concentrate feeds were found to have the highest digestibility values and ME concentration when compared to other types of feedstuffs. The differences in ME of various feedstuffs reflected different contents of fermentable carbohydrates and available N in cereals and protein supplements. While fermentable carbohydrates increase gas production, it has been reported that the addition of degradable N compounds to fiber rich feeds decreases gas production due to better or improved capturing of nutrients and higher production of microbial protein. The carbon source is diverted from gas to microbial protein synthesis [11]. Although the predictive ME values were found within the range of reported values for a large number of feedstuffs [20], yet some feedstuffs showed a significant variation in ME values. Normally, grains or their byproducts had higher ME levels than others except DORB. The values for commercially available protein sources were intermediate. Such differences in digestibility and ME values could be due differences in their chemical composition.

**Table-3:** IVGP, ME content, DM and OM digestibility, and PF of feedstuffs.

Feed	Total gas (ml/200 mg)	ME (MJ/kg DM)	TDMD (%)	TOMD (%)	PF
Grains					
Maize	65.54^b^±0.49	11.51	78.60^bc^±0.68	80.43^b^±1.13	2.51±0.14
Oat	62.91^a^±0.91	10.79	74.10^a^±0.59	76.95^a^±0.64	2.50±0.25
Wheat	68.79^b^±1.20	12.02	76.28^b^±0.43	78.99^b^±0.48	2.10±0.04
Barley	60.41^a^±0.75	10.11	72.31^a^±1.33	74.02^a^±1.44	2.40±0.08
Oilseed cakes/meals					
GNC	48.36^c^±0.85	9.44	69.52^c^±1.40	71.98^c^±1.03	3.71±0.40
MOC	45.31^b^±0.92	9.21	66.24^b^±1.19	69.80^b^±0.85	3.30±0.22
CSC	43.08^a^±0.27	7.71	64.72^a^±0.15	66.70^a^±0.15	2.90±0.17
SBM	50.96^d^±0.49	9.24	78.53^d^±0.70	80.67^d^±0.77	3.09±0.25
Agro-industrial by-products					
Wheat bran	48.99^c^±0.73	9.78	62.39^b^±1.79	64.15^b^±1.97	2.59^a^±0.26
DORB	43.87^b^±1.08	7.39	60.68^b^±0.84	63.50^b^±0.99	3.06^ab^±0.26
Rice polish	42.65^a^±0.38	7.41	60.28^b^±0.31	62.82^b^±0.94	3.69^b^±0.09
Wheat straw	25.52±0.31	4.65	38.54^a^±1.24	40.06^a^±1.38	2.45^a^±0.10
Leguminous green fodders					
Cowpea	41.45±0.83	8.05	60.95±1.34	61.98±1.57	3.33±0.13
Berseem	41.33±0.89	8.51	61.44±1.15	63.76±1.29	3.16±0.32
Non-leguminous green fodders					
Maize	38.89^c^±0.30	8.19	58.97^c^±1.49	60.55^c^±1.98	2.69±0.25
Oat	40.79^c^±0.74	8.34	56.72^c^±0.90	58.55^c^±0.78	2.63±0.13
Sorghum	32.61^a^±0.10	6.86	52.20^a^±1.13	54.88^b^±1.33	2.23±0.06
Pearl millet	36.19^b^±0.49	7.63	54.94^b^±0.88	56.85^a^±1.53	2.75±0.42

a,b,c,d Means bearing different superscripts in a column differ significantly (*p<0.05) IVGP=*In vitro* gas production, ME=Metabolisable energy, PF=Partitioning factor, DM=Dry matter, OM=Organic matter, TDMD=True dry matter digestibility, TOMD=True organic matter digestibility, GNC=Groundnut cake, MOC=Mustard oil cake, CSC=Cotton seed cake, SBM=Soybean meal, DORB=Deoiled rice bran

## Conclusion

The energy feeds showed a great variation in chemical composition and ME content. The results of this study demonstrated that the kinetics of gas production of energy feed sources differed among themselves. Based on this study, high fermentative abilities of energy feeds for use in ruminant feeding ranked from the highest to the lowest were; maize, wheat, oat, barley, GNC, SBM, MOC, CSC, respectively. Evaluation of various feedstuffs is helpful in balanced ration formulation for field animals and under farm conditions for better utilization of these commonly available feed resources.

## Authors’ Contributions

CD and SSK formulated the experimental design. DK and LKD carried out the experimental work. DK wrote the manuscript and did the data analysis. All authors read the final manuscript and approved it.
